# Fusion of RR Interval Dynamics and HRV Multidomain Signatures Using Multimodal Neural Models for Metabolic Syndrome Classification

**DOI:** 10.3390/medsci14020197

**Published:** 2026-04-14

**Authors:** Miguel A. Mejia, Oscar J. Suarez, Gilberto Perpiñan, Leiner Barba Jimenez

**Affiliations:** 1 Faculty of Electronic Engineering, Popular University of Cesar, Valledupar 200001, Cesar, Colombia; miguelmejia@unicesar.edu.co (M.A.M.); ivan.iseda@gmail.com (G.P.); 2 Faculty of Engineering and Architecture, University of Pamplona, Km 1 Bucaramanga Road, University Campus, Pamplona 543050, Norte de Santander, Colombia; 3 Mechatronics Engineering Department, Faculty of Engineering and Architecture, University of Pamplona, Km 1 Bucaramanga Road, University Campus, Pamplona 543050, Norte de Santander, Colombia; 4 Optic and Computer Science Laboratory, Popular University of Cesar, Valledupar 200001, Cesar, Colombia; barba.leiner@unicesar.edu.co

**Keywords:** RR interval dynamics, heart rate variability (HRV), metabolic syndrome, oral glucose tolerance test (OGTT), convolutional neural network (CNN), support vector machine (SVM), long short-term memory (LSTM)

## Abstract

**Background:** Metabolic syndrome (MetS) leads to alterations in cardiac autonomic control that can be detected from electrocardiogram (ECG)-derived markers, particularly when the cardiovascular system is challenged during an oral glucose tolerance test (OGTT). **Methods:** In this paper, we present an automated framework for MetS identification using RR intervals and heart rate variability (HRV) features extracted from 12-lead ECG recordings acquired during the five OGTT stages in 40 male participants (15 with MetS, 10 controls, and 15 endurance-trained marathon runners). RR intervals were first derived using a multilead Pan-Tompkins approach with fusion-based validation. From these RR series, HRV descriptors were computed from time-domain statistics (RR mean, SDNN, rMSSD, pNN50), spectral indices (VLF, LF, HF, LF/HF), and nonlinear measures (SD1, SD2, SampEn, DFA-$\alpha_{1}$). Conventional HRV analysis revealed pronounced physiological differences between groups: MetS subjects exhibited reduced parasympathetic activity, reflected by lower rMSSD and SD1, lower HF power, and higher LF/HF ratios, whereas marathoners showed greater vagal modulation, higher HF power, and increased signal complexity. Healthy controls showed an intermediate autonomic profile. Using RR sequences and HRV descriptors (256 samples per stage), we trained three multimodal classifiers: a CNN-MLP model with a softmax output, a CNN-MLP model with an SVM head, and a CNN + LSTM-MLP + SVM architecture. **Results:** All models achieved strong discriminative performance, with accuracies ranging from 0.92 to 0.95, F1-macro values from 0.92 to 0.95, and macro-AUC values from 0.96 to 0.97. The CNN-MLP model achieved the best overall performance, whereas the CNN + LSTM-MLP + SVM model showed strong class discrimination, particularly for endurance athletes, while maintaining competitive recall for MetS. **Conclusions:** These findings support the feasibility of ECG-based autonomic assessment as a complementary non-invasive approach for early metabolic risk detection in clinical and preventive cardiometabolic screening settings.

## 1. Introduction

Heart rate variability (HRV), obtained from beat-to-beat oscillations in consecutive RR intervals, has long been recognized as a noninvasive biomarker of autonomic cardiac regulation [1,2]. There is a considerable amount of evidence showing that reductions in HRV are associated with impaired metabolic control, chronic inflammation, insulin resistance, and increased risk of cardiovascular disease. Over the last decade, a number of well-designed studies have solidified this association [3,4]. Constant comparisons consistently show that patients with metabolic syndrome (MetS) exhibit reduced vagal modulation, reduced global variability, and nonlinear HRV, reflecting autonomic rigidity [3,4]. Supporting data from controlled metabolic challenges suggest that autonomic dysfunction may also develop in individuals with nondiabetic conditions showing excessive glucose excursions after ingestion [5]. These findings collectively establish HRV as an early indicator of cardiovascular and cardiometabolic impairment. They provide an informative physiological contrast in endurance-trained individuals.

Higher-intensity or prolonged bouts of exercise are often associated with dramatically increased HRV, a more prominent parasympathetic profile, and improved autonomic flexibility. Current evidence in athletes suggests retained complexity, increased dynamic stability of RR intervals, and improved recovery of autonomic modulation following metabolic load [6]. These features delineate an individual autonomic phenotype, providing useful information on how autonomic regulation adapts to different metabolic scenarios when compared with those within MetS and healthy control cohorts.

The oral glucose tolerance test (OGTT) serves as a rigorous experimental stimulus to evaluate such adaptations. The OGTT, as a standardized metabolic stimulus, consistently elicits coordinated autonomic, endocrine, and vascular responses [7,8]. An interest in characterizing cardiac autonomic behavior during the OGTT has also greatly expanded. Recent studies show that, through neurohumoral pathways, transient increments in glucose and insulin regulate RR dynamics [6,9]. These regulatory responses are often blunted in MetS [10], whereas endurance-trained marathoners exhibit a robust parasympathetic rebound and accelerated re-stabilization of autonomic activity. These patterns suggest that the responses of the RR interval during an OGTT reflect subtle disturbances in neurocardiac control that remain undetectable under resting conditions. However, despite this diagnostic potential, relatively few studies have assessed autonomic regulation in all stages of OGTT, and even fewer have described dynamic HRV activity throughout the protocol.

Relatively short HRV indices capture relevant yet fundamental aspects of autonomic control, but fall short of providing the complete temporal organization and dynamic architecture of RR interval behavior [9]. Therefore, recent analytical frameworks increasingly use nonlinear and complexity-based metrics, including entropy measurements, fractal scaling exponents, symbolic dynamics, and Poincaré descriptors, which have been shown to contribute to the early detection of metabolic dysregulation and cardiometabolic risk [9]. However, HRV descriptors may miss transient or non-stationary patterns that develop solely during the metabolic stimulation period. This constraint has driven the rise of integrative techniques that combine traditional HRV metrics, nonlinear dynamics, and time-series analysis.

Artificial intelligence developments have expedited this transition. Deep learning approaches, especially convolutional neural networks (CNNs), have achieved a level of expert performance in arrhythmia detection, ECG morphology measurement, and multilead cardiac classification [11,12]. Long short-term memory (LSTM) networks and gated recurrent units can also leverage long-range dependencies and autonomic oscillations in physiological time-series [13,14,15,16,17]. More recent hybrid architectures, such as CNN-LSTM architectures, attention mechanisms, and transformer-based models, have helped advance the characterization of autonomic state, stress reactivity, and cardiometabolic risk [18]. Nonetheless, the majority of available studies analyze RR interval sequences and heart rate variability (HRV) descriptors in isolation. Integrative multimodal approaches remain scarce; only a limited number of studies have applied deep learning frameworks to metabolic challenge paradigms, such as the OGTT test [19]. Furthermore, many studies continue to employ record-wise validation, a practice recognized to introduce data leakage and yield inflated performance estimates [20].

Based on the above limitations, this paper proposes a multimodal deep learning framework that combines knowledge from RR timing series with HRV descriptors from the time, frequency, and nonlinear domains across the five stages of OGTT. We assess three architectures for increasing complexity, which are (i) a convolutional neural network with a multilayer perceptron, (ii) the same convolutional encoder along with a support vector machine (SVM), and (iii) a temporal architecture with long short-term memory layers augmented with an SVM classifier. An 80/20 subject-wise split was used, with 80% allocated to training and internal validation and 20% to unbiased testing. This multimodal construction can facilitate the joint modeling of RR dynamics and multidomain HRV descriptors during the OGTT and the study of the extent to which representations reflect autonomic modifications across different metabolic states. The subject-wise evaluation approach also guarantees sound generalization estimates and addresses the data leakage problem typical in record-wise validation. The present framework integrates temporal data of RR intervals with the physiological content embedded in HRV characteristics, thereby yielding a more comprehensive characterization of autonomic responses in MetS, healthy individuals, and endurance athletes. This suggests that multimodal fusion facilitates the discrimination of metabolic dysfunction beyond what either modality alone can achieve.

## 2. Materials and Methods

### 2.1. Study Population and Protocol

Based on clinical and functional criteria, forty adult male volunteers participated in this study. Fifteen participants were diagnosed with metabolic syndrome (MetS) according to the NCEP ATP III guidelines [21]. The control group (C) consisted of ten healthy individuals, and the remaining fifteen participants were endurance-trained marathon runners (M) who typically ran between 180 and 240 km per week. Participants were recruited from individuals undergoing routine clinical evaluation at the University Hospital of Caracas.

Endurance-trained marathon runners were included as a physiological reference group, in addition to participants with metabolic syndrome and healthy controls, as they exhibit enhanced autonomic regulation associated with long-term aerobic training [22,23]. This design enabled the comparison of three different autonomic profiles spanning a continuum of autonomic regulation, ranging from impaired modulation in metabolic syndrome to enhanced vagal modulation in endurance-trained athletes. Participants were excluded if they had known cardiovascular disease, arrhythmias, or ECG abnormalities that could significantly interfere with reliable RR interval analysis, diabetes mellitus or other metabolic disorders different from metabolic syndrome, neurological diseases affecting autonomic regulation, or treatment with medications known to influence heart rate variability, such as beta blockers. To reduce potential confounding effects on autonomic measurements, participants were instructed to refrain from caffeine, alcohol consumption, and intense physical exercise prior to the recordings.

All participants underwent a standardized oral glucose tolerance test (OGTT) after an overnight fast. Venous blood samples were obtained at five time points corresponding to baseline (0 min), 30 min, 60 min, 90 min, and 120 min following the ingestion of a 75 g glucose load. At each stage, glucose, insulin, triglycerides, and HDL cholesterol levels were measured. Immediately after each blood draw, a resting 12 lead ECG was recorded for at least 10 min at a sampling frequency of 1000 Hz and a resolution of 16 bits. Thus, five ECG recordings were obtained for each participant, corresponding to the five OGTT stages, yielding a total of 200 ECG recordings (40 subjects × 5 stages).

All procedures were conducted in accordance with the ethical principles of the Declaration of Helsinki and its subsequent amendments. The recruitment procedures, informed consent process, and data acquisition protocol have been previously described in a peer-reviewed IEEE publication [24]. Table 1 presents the clinical and biochemical characteristics of the three study groups. Participants with MetS exhibited higher body mass index, blood pressure, triglyceride concentrations, and fasting and post-load glucose and insulin levels compared with the control group and marathon runners. In contrast, marathon runners showed lower fasting and post-load glucose and insulin concentrations, as well as reduced area under the curve values, indicating enhanced metabolic efficiency.

### 2.2. Signal Processing

All ECG recordings were processed using a structured, transparent, and fully reproducible signal processing pipeline designed to ensure the consistent detection of cardiac events and reliable characterization of autonomic modulation. We first extracted R peaks independently from the 12- leads using a modified implementation of the Pan-Tompkins algorithm, one of the most widely validated approaches for real-time QRS detection [25]. Each channel was bandpass-filtered (5–15 Hz) using a third-order Butterworth filter to attenuate baseline drift and high-frequency artifacts [26]. The filtered signals were then differentiated to enhance rapid QRS transitions, squared to amplify steep slopes, and integrated using a moving window operator to obtain an energy envelope highlighting candidate QRS complexes.

Initial peak detections were obtained through adaptive thresholding applied to the integrated signal. To avoid multiple detections within a single cardiac cycle, a physiological refractory period of 200 ms was enforced during peak detection. Each detected peak was subsequently refined by locating the maximum amplitude within a local window of ±50 ms around the candidate peak in the filtered ECG signal.

To improve robustness, particularly under noise, motion artifacts, or morphological variability across leads, detections from the twelve ECG channels were combined using a multichannel fusion strategy. Candidate R peaks detected independently in each lead were temporally aligned and grouped using a clustering tolerance of 50 ms. The final R peak position was defined as the median timing of the clustered detections, producing a fused R-peak sequence that integrates information from all leads while suppressing spurious detections.

**Figure 1:**
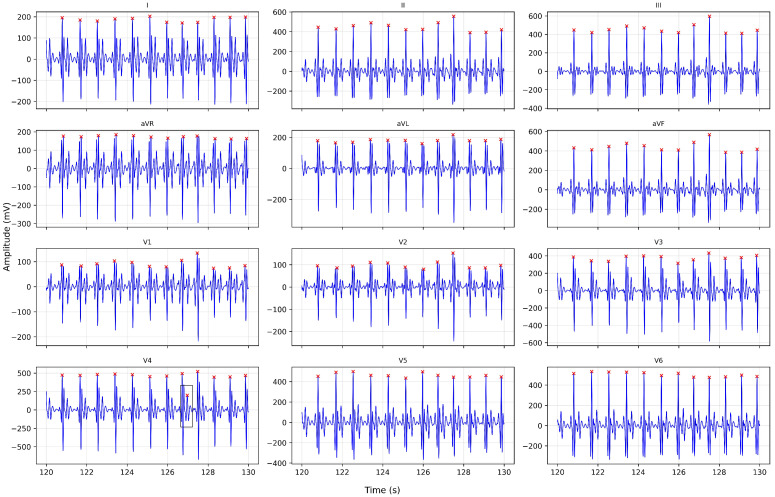
Ten-second segment of a twelve-lead ECG excerpt in which detected R peaks are shown in red. The multichannel fusion mechanism removes a spurious event in lead V4 and enables a consistent, physiologically meaningful identification of cardiac cycles.

Figure 1 presents an example of the multichannel detection approach applied to a ten-second segment of a twelve-lead ECG. The red markers identify the R peaks detected in each channel, and an isolated artifact visible in lead V4 is excluded by the fusion mechanism. This outcome demonstrates that integrating information from all leads enhances the robustness of the detection process and supports a more physiologically consistent identification of cardiac cycles.

After multichannel fusion, RR interval time-series were constructed from the fused R peak positions. RR intervals were defined as the temporal difference between consecutive R peaks and expressed in milliseconds. The resulting RR series was subsequently inspected for abnormal intervals and artifacts. Outliers were identified using a moving median filter with a window size of five beats. RR intervals deviating more than 20% from the local median were considered artifacts and removed from the sequence.

Missing or corrupted RR intervals were then corrected using linear interpolation in order to obtain a continuous RR time-series suitable for variability analysis. For each OGTT stage, contiguous RR segments were selected for analysis. Short-term HRV analysis is typically conducted using segments of approximately five minutes, which are considered sufficient for reliable estimation time domain, frequency domain, and nonlinear indices of autonomic modulation. Five-minute recordings represent the standard duration for short-term HRV analysis and have been widely adopted in physiological and clinical studies, according to the recommendations of the Task Force of the European Society of Cardiology and the North American Society of Pacing and Electrophysiology [27,28]. In this study, each ECG recording lasted approximately ten minutes, allowing for the selection of artifact-free five- minute RR segments to ensure consistent HRV characterization across the different OGTT stages. In accordance with standard recommendations for short-term HRV analysis, five-minute RR segments were used to characterize autonomic modulation.

For the deep learning models, a sequence of 256 consecutive RR intervals was extracted from each segment to represent short-term heart rate dynamics and serve as input to the sequential branch of the multimodal classifier.

### 2.3. HRV Feature Extraction

Once the fused R peak positions were obtained, the RR interval series for all OGTT stages were analyzed using automated procedures that included visual inspection to confirm that the temporal patterns were physiologically plausible. Ectopic beats, artifacts, and intervals that deviated from the expected cardiac timing were identified using adaptive criteria based on local variability, slope changes, and median filtering. Only small patches that clearly required correction were interpolated, and this was restricted to the minimum number of points required to maintain the natural temporal structure of the signal. This cautious method preserved the nonlinear nature of the RR dynamics and minimized the risk of altering important features through excessive preprocessing.

An extensive selection of HRV descriptors for the five OGTT time points (0, 30, 60, 90, and 120 min) was then computed following a previously described protocol [29,30]. The features were categorized into three domains representing complementary dimensions of autonomic function. The time domain included mean RR, SDNN, RMSSD, pNN50, and SDSD, which are commonly used measures to quantify short- and long-term variability. The frequency domain consisted of VLF, LF, HF power, and the LF/HF ratio, all obtained using Welch spectral estimation to provide stable representations of oscillatory components. The nonlinear domain included Poincaré indices (SD1 and SD2), Sample Entropy (SampEn), Approximate Entropy (ApEn), and detrended fluctuation analysis coefficients (DFA-$\alpha_{1}$ and DFA-$\alpha_{2}$), which capture the complexity and self-similarity in cardiovascular regulation.

Collectively, these descriptors provide a comprehensive view of the dynamics emerging under autonomic control, incorporating rapid fluctuations, slower oscillatory components, and nonlinear regulatory behavior. In total, 32 HRV descriptors were computed for each OGTT stage. Prior to model training, HRV features were standardized using z-score normalization computed from the training dataset to ensure comparable scaling across subjects.

**Figure 2:**
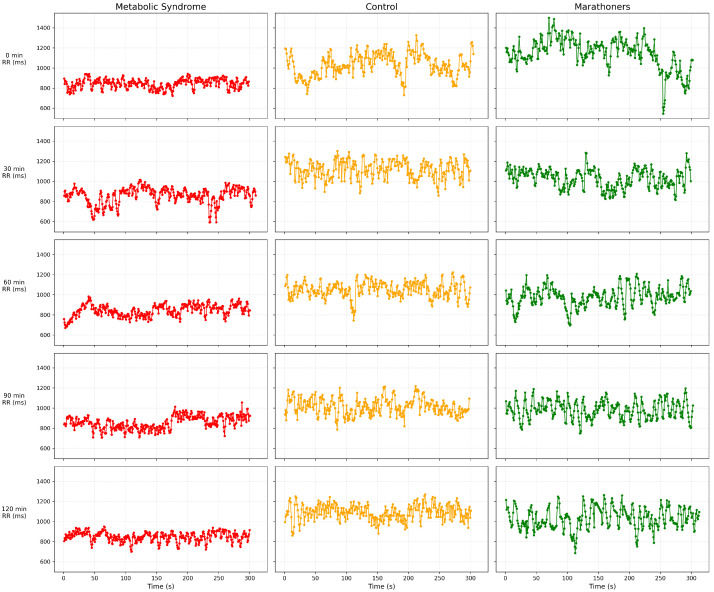
RR interval series (five-minute segments) recorded across the OGTT stages for one representative case from each group. Metabolic syndrome is shown in the red signal, the healthy control in the yellow signal, and the marathon runner in the green signal.

The RR interval trajectories for one representative participant from each study group are shown in Figure 2. As illustrated, the metabolic syndrome case displays reduced variability, healthy individuals show moderate autonomic modulation, and marathon runners exhibit broader and more adaptive fluctuations consistent with enhanced autonomic flexibility. The HRV parameters extracted at each stage are summarized in three tables in the Section 2, presenting median and interquartile range values for the time, frequency, and nonlinear domains. Differences between groups were evaluated using the Kruskal–Wallis test followed by Dunn’s multiple comparison tests when appropriate. Considering five OGTT stages per subject, the final dataset consisted of 200 multistage recordings (40 subjects × 5 stages), each represented by a multimodal pair composed of a 256-sample RR sequence and a 32-dimensional HRV feature vector.

### 2.4. Classification Methodology

#### 2.4.1. Model Development

To examine autonomic alterations during the OGTT, we implemented three supervised learning architectures that represent current strategies for analyzing biomedical time-series. The first architecture was a multimodal convolutional model that combined a convolutional encoder with a multilayer perceptron. The second architecture used the same convolutional and dense modules, but replaced the final softmax layer with a support vector machine classifier. The third architecture extended the convolutional front end by incorporating recurrent processing with long short-term memory units, together with a final support vector machine classifier.

These architectures were selected based on previous evidence showing that convolutional encoders achieve strong performance for ECG-based morphology learning [14,15,17]. Recurrent units, particularly long short-term memory layers, capture temporal dependencies that are relevant to autonomic modulation [13,16]. Furthermore, hybrid configurations that combine deep feature encoders with discriminative classifiers have demonstrated improved decision boundaries and enhanced generalization on moderate-sized biomedical datasets [13,15,17].

All models processed the RR interval sequences obtained using the multilead Pan-Tompkins procedure. The multimodal configurations also received a set of 32 standardized HRV descriptors that represented the time domain, frequency domain, and nonlinear dynamics. This design enabled the models to integrate the beat-to-beat morphology encoded in the RR sequence with the broader statistical and complex variability patterns captured by the HRV measures [9,29].

#### 2.4.2. Subject-Wise Data Partitioning

During model development, the dataset was divided into three independent subsets: training, validation, and test. To guarantee strict independence between participants and prevent information leakage across repeated measurements, the partitioning was performed at the subject level rather than at the sample level. Consequently, all RR interval sequences and HRV feature vectors belonging to the same participant were assigned exclusively to a single subset. This constraint is essential in physiological time-series analysis, where intra-subject correlations may otherwise lead to overly optimistic performance estimates.

A subject-wise hold-out strategy was adopted. First, the 80% of the participants were assigned to the development subset and the remaining 20% to the test subset. Then, within the development subset, an additional subject-wise split was performed to reserve 10% of the subjects for internal validation. This validation subset was used to monitor convergence and apply early stopping during training, while the test subset remained completely unseen until the final evaluation stage.

To further avoid data leakage, preprocessing steps applied to the HRV features, including missing-value imputation and standardization, were fitted exclusively on the training subset and subsequently transferred to the validation and test subsets. In addition, variables associated with subject identity were excluded prior to training. Final model performance was assessed on the held-out test subjects using accuracy, precision, sensitivity, specificity, and F1 score.

### 2.5. Model Architectures

**Figure 3:**
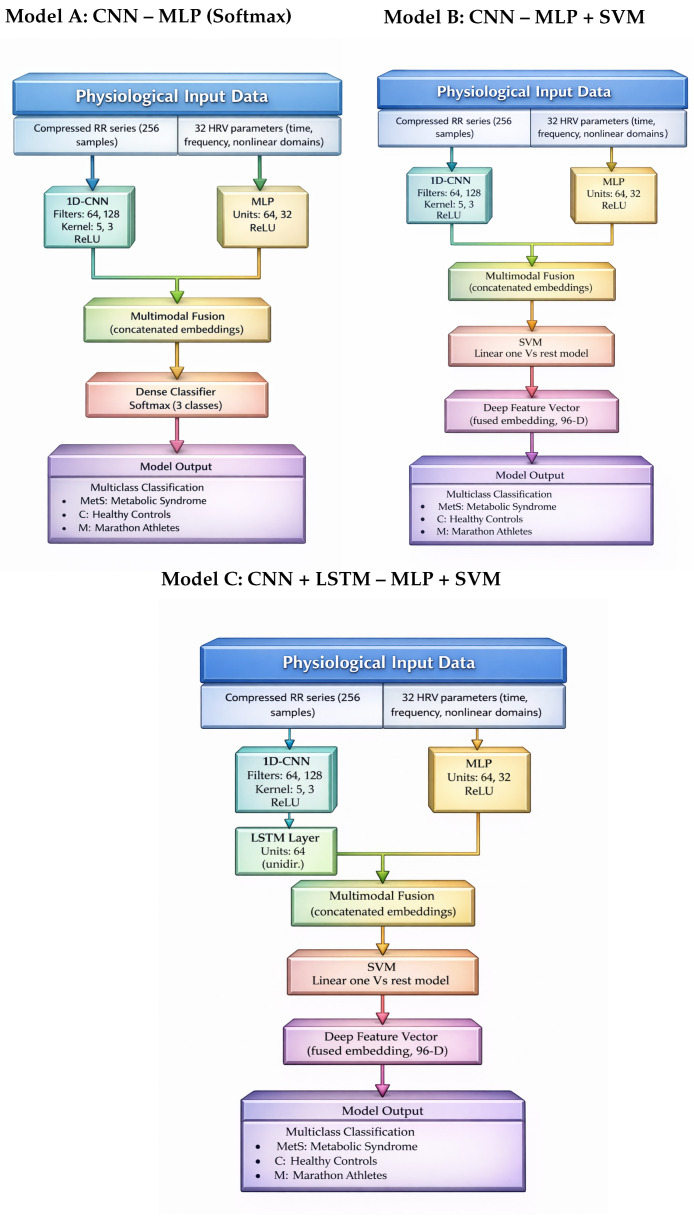
Comparison of the three supervised multimodal neural model architectures implemented for OGTT-based autonomic classification.

Three multimodal classification architectures, as can be seen in Figure 3, were designed to evaluate different strategies for integrating temporal RR interval dynamics with statistical heart rate variability (HRV) descriptors. All models receive two complementary inputs: (1) a temporal sequence of RR intervals and (2) a vector of handcrafted HRV features derived from the same recordings. The RR sequence was represented as a fixed-length vector of 256 samples obtained after preprocessing and interpolation, while the HRV branch consisted of a standardized 32-dimensional feature vector including time-domain, frequency-domain, and nonlinear metrics.

#### 2.5.1. Model A: CNN–MLP (End-to-End Multimodal Baseline)

Model A implements an end-to-end multimodal architecture in which RR interval sequences and HRV descriptors are jointly encoded and classified using a neural network. The RR stream is processed by a two-block one-dimensional convolutional encoder designed to capture short-range temporal structures in cardiac rhythm dynamics.

The first convolutional block consists of a 1D convolution layer with 64 filters (kernel size = 5, stride = 1, ReLU activation), followed by batch normalization, max pooling (pool size = 2), and dropout (rate = 0.2). The second convolutional block includes a 1D convolution layer with 128 filters (kernel size = 3, ReLU activation), batch normalization, max pooling, and dropout (rate = 0.2). The resulting feature maps are flattened to produce a learned RR embedding of 64 dimensions.

In parallel, the HRV branch receives a standardized 32-dimensional feature vector. These descriptors are processed through a multilayer perceptron with 64 and 32 neurons (ReLU activation), producing a 32-dimensional HRV embedding. The RR and HRV embeddings are then directly concatenated, implementing a late-fusion strategy that combines temporal features and global autonomic descriptors into a fused multimodal representation of 96 dimensions.

The fused embedding is then processed by a final classification layer with softmax activation to estimate the probability of the three classes: Metabolic Syndrome (MetS), Control (C), and Marathon Athletes (M).

Convolutional encoders similar to this architecture have demonstrated strong performance for ECG morphology learning and rhythm discrimination in large-scale studies [12,19]. Furthermore, combining deep-learned representations with handcrafted HRV descriptors has been shown to improve robustness when datasets are relatively small [10,11,20].

#### 2.5.2. Model B: CNN–MLP + SVM (Hybrid Deep Feature Extractor)

Model B shares the same multimodal feature encoder as Model A, but replaces the neural softmax classifier with a linear one-versus-rest Support Vector Machine (SVM). After supervised training of the CNN–MLP backbone, the latent representation obtained after fusion corresponds to a compact 96-dimensional multimodal feature vector.

This representation is subsequently used as input to an SVM classifier with a linear kernel. Hybrid deep feature extractor + SVM schemes have been widely adopted in biomedical signal analysis, where margin-based classifiers often improve decision boundaries and generalization when datasets are limited or moderately sized [13,14,19,31].

This configuration allows for the architecture to retain the expressive capacity of the deep convolutional encoder while leveraging the stability and strong generalization properties of SVM classifiers.

#### 2.5.3. Model C: CNN + LSTM–MLP + SVM (Temporal Contextual Multimodal Model)

Model C extends the previous architectures by incorporating explicit temporal modeling of RR interval dynamics. As in the previous models, the RR stream is first processed through the same two convolutional blocks (64 and 128 filters) used in Model A and Model B, producing a sequence of convolutional feature maps that encode local morphological patterns of the RR signal.

Instead of directly flattening these features, the resulting sequence is fed into a unidirectional Long Short-Term Memory (LSTM) layer with 64 hidden units. This recurrent layer allows for the model to capture medium-range temporal dependencies and autonomic fluctuations that cannot be fully represented by convolutional operators alone.

The temporal embedding generated by the LSTM layer is then concatenated with the 32-dimensional HRV embedding, again using late multimodal fusion. The resulting fused representation has 96 dimensions and is subsequently used for classification through a linear one-versus-rest SVM classifier.

Hybrid CNN–LSTM architectures have consistently demonstrated state-of-the-art performance in ECG sequence modeling and physiological time-series analysis [13,19,32]. Similarly, deep encoders combined with SVM classifiers have shown improved discriminative capability in ECG-based disease detection tasks [33].

### 2.6. Statistical Hypothesis Tests

We performed statistical hypothesis tests with a 5% significance level to evaluate differences in HRV parameters among the three study groups across the OGTT stages. Prior to group comparisons, normality was assessed using the Shapiro–Wilk test, which indicated that most variables did not follow a Gaussian distribution. Therefore, all subsequent analyses were conducted using nonparametric methods.

Group comparisons at each OGTT stage were performed using the Kruskal–Wallis test for independent samples. When the null hypothesis was rejected, pairwise differences were examined using Dunn’s post hoc test with Bonferroni adjusted *p* values.

All resulting *p*-values obtained for the time, frequency, and nonlinear HRV parameters are presented in Table 2, Table 3 and Table 4. These tables provide a consolidated view of the statistical comparisons across the OGTT stages and study groups.

### 2.7. Evaluation Metrics

The behaviour of the proposed classifiers was assessed using a confusion matrix, which summarizes the counts of true positives (TP), true negatives (TN), false positives (FP), and false negatives (FN) for each class. From these quantities, we derived a set of widely used performance indicators in biomedical signal classification, including accuracy (Acc), sensitivity ($S_{e}$), specificity ($S_{p}$), positive and negative predictive values (PPV and NPV), and the false positive and false negative rate (FPR and FNR). These metrics are recommended for multiclass physiological classification tasks, especially when class imbalance or substantial inter-subject variability is present [34,35,36].

The false positive and false negative rates, denoted as FPR and FNR, were computed using Equations (1) and (2):

(1) $$\mathrm{FPR}=\frac{FP}{FP+TN},$$

(2) $$\mathrm{FNR}=\frac{FN}{TP+FN}.$$

Predictive values and class-wise sensitivity and specificity, denoted as PPV, NPV, $S_{e}$, and $S_{p}$, were obtained using Equations (3)–(6):

(3) $$\mathrm{PPV}=\frac{TP}{TP+FP},$$

(4) $$\mathrm{NPV}=\frac{TN}{TN+FN},$$

(5) $$S_{e}=\frac{TP}{TP+FN},$$

(6) $$S_{p}=\frac{TN}{TN+FP}.$$

Overall accuracy, denoted as Acc, which aggregates all confusion matrix components into a single global indicator, was expressed as shown in Equation (7):

(7) $$\mathrm{Acc}=\frac{TP+TN}{TP+TN+FP+FN}.$$

Beyond these confusion matrix-derived measures, we also quantified macro and weighted F1 scores, balanced accuracy, Cohen’s κ, and the Matthews correlation coefficient (MCC), all of which provide complementary insights into multiclass discrimination performance [34]. The area under the receiver operating characteristic curve (AUROC) was computed under a one vs rest (OvR) formulation, reporting both macro- and micro-averaged values. For the HRV feature branch of the multimodal classifier, feature importance was estimated using SHAP-based gradient explanations, enabling the assessment of the relative contribution of each HRV descriptor to the model’s predictions.

## 3. Results

A total of 40 participants completed the five stages of the OGTT, providing high-quality RR interval time-series for HRV analysis and enabling the construction of multimodal classification models. In this section, we first describe group differences in HRV parameters across OGTT stages, and then present the performance of multimodal deep learning classifiers for three-class discrimination between metabolic syndrome (MetS), healthy controls (C), and marathoners (M). Unless otherwise stated, values are reported as medians (interquartile ranges), and between-group comparisons correspond to Kruskal–Wallis tests with post hoc pairwise contrasts.

### 3.1. Time Domain HRV Parameters

As shown in Table 2, subjects with MetS exhibited consistently shorter mean RR intervals across all OGTT stages compared with marathoners, with statistically significant differences at every time point (all *p* values < 0.01). This pattern reflects a higher resting heart rate and reduced vagal modulation, in line with population-based studies linking metabolic dysregulation to diminished HRV amplitude and parasympathetic withdrawal [4,9]. Differences between the MetS and control groups were more modest; nevertheless, a significant reduction in mean RR was observed at the 30 min stage (*p* = 0.014), suggesting an accentuated chronotropic response to the early glucose load in the MetS group. At baseline, SDNN was significantly lower in MetS than in controls (47.6 vs. 91.0 ms, *p* = 0.040), indicating reduced global HRV under resting conditions, which agrees with previous evidence of overall variability loss in individuals with clustered metabolic risk factors [3,9]. Across the remaining OGTT stages, no statistically significant group differences were found for RMSSD, SDSD, or pNN50, suggesting that short-term beat-to-beat variability is primarily altered at rest rather than during the acute metabolic challenge. Endurance athletes consistently showed longer RR intervals and higher variability indices (SDNN, RMSSD, SD1) than the other groups, consistent with the enhanced parasympathetic tone and improved autonomic efficiency described in trained populations [37]. Overall, time domain results indicate that autonomic impairment in MetS is most evident at baseline, characterized by shorter RR intervals and lower global variability. In contrast, OGTT-induced changes in short-term indices show limited discriminatory power between groups.

### 3.2. Frequency Domain HRV Parameters

Table 3 summarizes the frequency domain HRV results across the study groups and OGTT stages. At the baseline measurement, notable contrasts appeared in the very low-frequency (VLF) and low-frequency (LF) ranges. Individuals with MetS displayed substantially lower VLF power than both marathoners (*p* = 0.036) and healthy controls (*p* = 0.016). This reduction in slower oscillatory components agrees with previous work linking MetS to weakened baroreflex activity and a decline in long-period autonomic rhythms [3,4,10]. LF power also tended to be lower in MetS compared with healthy controls (*p* = 0.047), suggesting a reduced oscillatory contribution to autonomic regulation.

The LF/HF ratio at baseline was higher in healthy controls than in MetS and Marathoners groups, a trend consistent with the sympathetic predominance often observed in sedentary adults [30]. Marathoners, on the other hand, exhibited lower LF/HF values, reflecting the vagally oriented autonomic profile characteristic of endurance training [37].

When the remaining OGTT stages were examined, the frequency-based measures did not reveal statistically significant differences among the groups, indicating that the short-term spectral adjustments following glucose ingestion were broadly comparable. Taken together, the baseline spectral indicators provided stronger group separation than the dynamic changes observed during the glucose challenge. These findings suggest that the autonomic alterations associated with MetS become more pronounced under resting conditions, where reductions in global spectral power and weakening of low-frequency rhythms are most evident.

### 3.3. Nonlinear HRV Parameters

Table 4 presents the comparison of nonlinear HRV descriptors across groups and OGTT stages. At baseline, significant differences were observed in the short-term fractal scaling exponent DFA-$\alpha_{1}$. Participants with MetS showed lower values than both controls (*p* = 0.017) and marathoners (*p* = 0.028). Reduced DFA-$\alpha_{1}$ indicates a shift toward more random short-term dynamics and diminished fractal organization, a pattern frequently reported in cardiometabolic dysfunction [3,4].

Baseline differences were also evident in long-range variability indices. SD2, which reflects broader oscillatory behaviour in the Poincaré plane, was significantly lower in MetS than in controls (*p* = 0.022). This reduction has been linked to impaired baroreflex engagement and decreased autonomic adaptability in metabolic disorders [4]. Conversely, the SD1 to SD2 ratio was higher in MetS (*p* = 0.003), suggesting a compression of long-term variance relative to short-term fluctuations, consistent with early autonomic impairment [9].

Entropy-based measures showed fewer baseline differences. Approximate entropy and sample entropy tended to be lower in MetS, indicating reduced irregularity and diminished dynamical richness in the cardiac rhythm. These metrics were originally proposed as markers of complexity and physiological variability in cardiovascular time-series [38]. Notably, sample entropy at the 120 min stage was significantly lower in MetS than in marathoners (*p* = 0.041) and controls (*p* = 0.024). These late-stage differences may reflect group specific autonomic responses to the progressive metabolic load during the OGTT, consistent with the sensitivity of entropy indices to metabolic and inflammatory stressors.

Across OGTT stages, nonlinear metrics did not show consistent differences among groups, suggesting that resting-state complexity markers are more informative than acute glucose responses. Overall, the nonlinear descriptors reveal that MetS is characterized by reduced fractal organization, diminished long-term variability, and lower system irregularity, features that reflect impaired autonomic complexity and early metabolic dysregulation [9].

### 3.4. Multimodal Deep Learning Models for Metabolic Syndrome Classification

A total of 200 multistage recordings were used for model development, corresponding to five OGTT stages (baseline, 30, 60, 90, and 120 min) from 40 subjects. Each sample consisted of two complementary inputs: (i) a short RR interval sequence describing beat-to-beat dynamics and (ii) a vector of 32 handcrafted HRV descriptors derived from time, frequency, and nonlinear domains. HRV features were standardized using z-score normalization computed exclusively on the training data.

Three multimodal neural architectures were evaluated to integrate sequential RR dynamics with HRV descriptors. The RR branch was modeled using a one-dimensional convolutional neural network (1D-CNN) that processes the input sequence $x\in R^{256\times 1}$. The architecture includes two convolutional blocks composed of Conv1D layers (64 and 128 filters; kernel sizes 5 and 3), ReLU activations, and MaxPooling (pool size = 2). The resulting representation is projected to a 64-dimensional embedding $z_{RR}$.

The HRV branch processes the standardized feature vector $h\in R^{32}$ using a multilayer perceptron with two dense layers (64 and 32 neurons, ReLU activation), producing a 32-dimensional embedding $z_{HRV}$.

Both modalities are integrated using late multimodal fusion via vector concatenation,

$$z_{\mathrm{fusion}}=\left[z_{RR},z_{HRV}\right]\in R^{96},$$

which serves as the joint representation for classification.

Based on this shared encoder, three classifier configurations were investigated:

1. **CNN–MLP**: The fused representation is classified using a fully connected layer with Softmax activation for three-class prediction (MetS, Control, Marathon).
2. **CNN–MLP + SVM**: The multimodal encoder acts as a feature extractor and the fused embedding is classified using a linear Support Vector Machine.
3. **CNN + LSTM–MLP + SVM**: An additional LSTM layer (64 units) is included in the RR branch to model temporal dependencies before multimodal fusion, with the fused representation classified using an SVM.

Models were trained using the Adam optimizer ($10^{-3}$ learning rate) and categorical cross-entropy loss. Training was performed with a batch size of 32 for up to 100 epochs with dropout regularization (0.2 in the convolutional blocks and 0.3 in the MLP layers). To prevent data leakage, dataset partitioning was performed at the subject level so that all recordings from a participant were assigned exclusively to either the training or testing set.

The main architectural parameters and training configuration are summarized in Table 5.

#### 3.4.1. Global Performance Across Models

Table 6 provides an overview of the global performance achieved through the independent test set with respect to the three class problem (MetS, C, and M). All models showed high discriminative quality, with macro F1 scores ranging from 0.92 to 0.95 and discriminative quality ranging from 0.92 to 0.95. All three indicate that the multimodal encoders perform well and are generalizable, despite their modest data size.

The CNN–MLP with a Softmax output layer achieved the best performance in terms of accuracy (0.95), macro F1 score (0.95), and strong agreement metrics (Cohen’s kappa = 0.92, MCC = 0.93).

The CNN–MLP + SVM was the second configuration, with an accuracy of 0.93, a macro F1 score of 0.92, and balanced accuracy and reliability metrics greater than 0.89. This similarity reaffirms that most of the discriminative power comes from the shared multimodal encoder rather than the classifier head.

The performance on the CNN + LSTM–MLP + SVM architecture was competitive, with an accuracy of 0.92 and a macro F1 score of 0.92. It is slightly less accurate than the fully convolutional models, but the AUC was 0.97, indicating excellent class separability across varying decision thresholds.

Overall, all three multimodal approaches yielded consistent, physiologically coherent results, with the CNN-MLP configuration being the most balanced, while the CNN + LSTM–MLP + SVM architecture proved to be a powerful discriminator in terms of AUC.

#### 3.4.2. Per Class Performance

Table 7 reports the precision, recall, and F1 score per class for each architecture in the test set. In the CNN-MLP model, the control group achieved perfect recall (1.00) with high precision (0.91), while marathoners achieved high precision and perfect recall (F1 = 0.97). The MetS class achieved perfect precision (1.00), but slightly reduced recall (0.87), indicating that the few errors primarily involved MetS subjects misclassified as healthy controls. Clinically, this behavior is conservative, as it minimizes false positive MetS predictions.

The CNN-MLP + SVM model reproduced an almost identical per class pattern, confirming that the discriminative strength arises mainly from the shared multimodal encoder rather than the classifier head. The CNN + LSTM–MLP + SVM architecture also produced strong class-wise performance, though with a moderate reduction in recall for the control (0.80) and MetS (0.87) groups. In contrast, marathoners remained consistently well classified across all models, suggesting that their autonomic profiles during the OGTT are the most distinct.

Overall, the integration of RR dynamics and HRV descriptors enabled reliable separation not only between MetS and non-syndromic subjects, but also between healthy controls and endurance athletes, demonstrating that subtle autonomic differences across groups can be effectively captured through multimodal ECG analysis.

#### 3.4.3. Contribution of HRV Parameters

In the HRV branch of multimodal models, the focus of importance analysis was on the most compact category of variables, comprising those that contributed more to the final decision (Table 8). These three architectures ranked similarly, exhibiting the influence of global variability indices (mean RR and SDNN), sympathovagal balance (LF/HF ratio), geometric descriptors extracted from the Poincaré plot (SD1/SD2 and ellipse area *S*), and nonlinear complexity measures (sample entropy and DFA-$\alpha_{1}$).

It shows that the models take advantage of physiologically interpretable HRV patterns that jointly encode overall variability, frequency band modulation, and signal complexity. When combined with the temporal information in the RR sequence, these descriptors provide complementary views of autonomic regulation, thereby supporting consistent discrimination among individuals with MetS, controls, and marathoners.

#### 3.4.4. Graphical Representation

**Figure 4:**
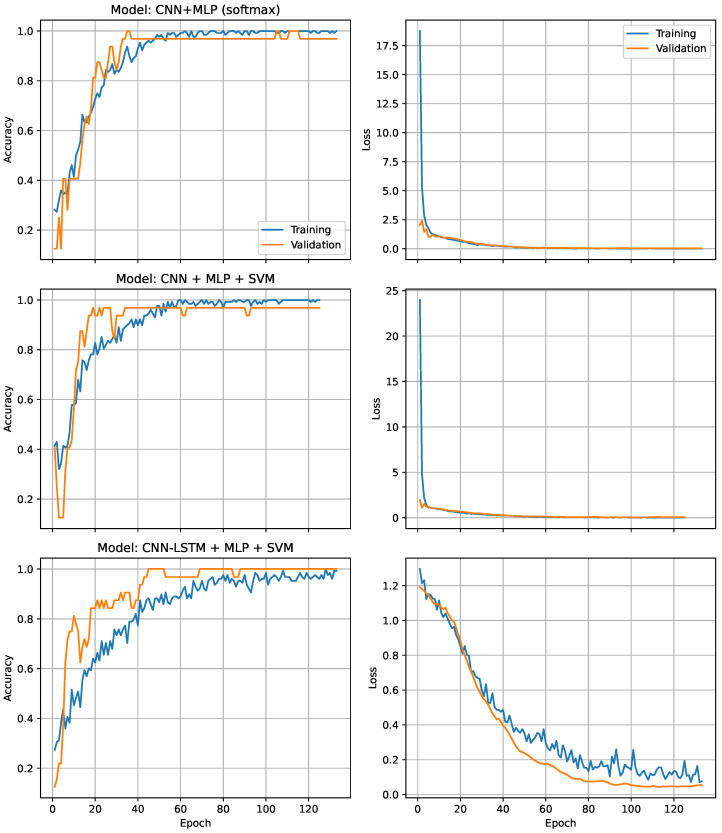
Training and validation accuracy and loss for the three multimodal architectures. From top to bottom: Model A (CNN–MLP), Model B (CNN–MLP + SVM), and Model C (CNN + LSTM–MLP + SVM).

Figure 4 summarizes the learning dynamics of the three multimodal architectures. Across all models, both training and validation curves exhibit rapid convergence during the first epochs, followed by stable behavior without divergence between loss trajectories. This pattern is consistent with controlled model capacity and adequate regularization, indicating that none of the architectures experienced marked overfitting. The CNN + LSTM–MLP backbone shows the smoothest validation loss profile, suggesting enhanced temporal generalization due to its recurrent component.

**Figure 5:**
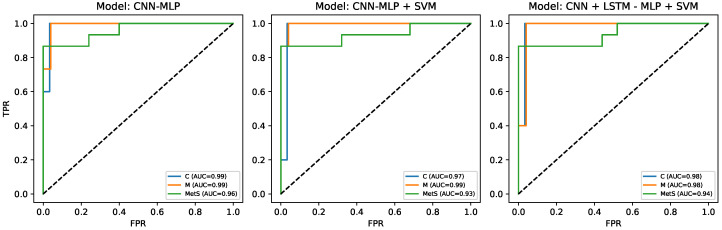
Receiver operating characteristic (ROC) curves for the three classes (C, M, and MetS) across the three multimodal models. The reported AUC values confirm strong class separability in all architectures.

Receiver operating characteristic (ROC) curves for all classes (Control, Marathoner, and MetS) and all architectures are shown in Figure 5. In all three models, the ROC curves consistently remain close to the upper left region of the plot, with class-wise AUC values ranging approximately from 0.93 to 0.99. Control and Marathoner profiles achieve the highest AUCs (up to 0.99), whereas the MetS class exhibits slightly lower but still excellent AUCs (0.93–0.96), reflecting a more heterogeneous autonomic phenotype. These results align with the global performance metrics reported in Table 6 and confirm strong separability among autonomic profiles across OGTT stages.

**Figure 6:**
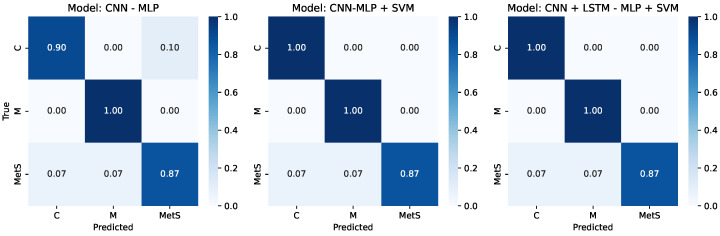
Normalized confusion matrices for the three multimodal models. Top: Model A (CNN–MLP); middle: Model B (CNN–MLP + SVM); bottom: Model C (CNN + LSTM–MLP + SVM). Values represent normalized recall per class.

To complement these analyses, Figure 6 displays the normalized confusion matrices for the three multimodal models. All configurations consistently show high recall for the Marathoner class, while the CNN-MLP + SVM and CNN + LSTM-MLP + SVM models achieve perfect classification of the Control group. The remaining misclassifications predominantly affect subjects with MetS, who are occasionally assigned to the Control or Marathoner classes, in line with the partial physiological overlap between these groups. Taken together, these visual results reinforce the high discriminative capacity of the proposed multimodal architectures and the added value of integrating RR dynamics with multidomain HRV features.

## 4. Discussion

MetS was associated with an altered cardiac autonomic profile characterized by reduced vagal control, consistent with established physiological models and prior evidence. Participants with MetS exhibited significantly lower vagally mediated HRV indices, specifically *rMSSD* and *SD1*, together with reduced HF power and a relatively higher LF/HF ratio. Collectively, these findings support diminished parasympathetic modulation and a relative sympathovagal imbalance. This interpretation is consistent with previous evidence showing that MetS is associated with lower short-term HRV and reduced autonomic flexibility [4,10,39].

To place these findings in context, several previous studies have systematically examined the relationship between MetS and HRV using observational, systematic review, and meta-analytic approaches. Overall, the available evidence consistently supports a reduction in vagally mediated HRV indices, lower global variability, and broader impairment in autonomic regulation among individuals with MetS. This convergence reinforces the interpretation that the autonomic alterations observed in the present study are not isolated findings, but rather part of a reproducible cardiometabolic phenotype [4,10,39].

In contrast, endurance athletes demonstrated greater autonomic efficiency, evidenced by higher HF power, increased nonlinear complexity (*SampEn*), and preserved *DFA-α1* dynamics in our cohort. The higher vagally mediated profile observed in athletes is in agreement with prior work showing that aerobic fitness and endurance training are associated with more favorable cardiac parasympathetic regulation and improved autonomic adaptation [40,41]. Healthy participants exhibited an intermediate autonomic profile between the MetS and athlete groups.

Furthermore, individuals with MetS displayed significantly lower VLF and LF power compared with controls, suggesting attenuation of longer-period oscillatory rhythms and possible impairment in baroreflex related cardiovascular modulation. This interpretation is supported by evidence indicating that metabolic syndrome is accompanied by neuroadrenergic and reflex abnormalities, including sympathetic activation and altered baroreflex control [42]. From a physiological perspective, these baseline alterations are compatible with impaired cardiovascular autonomic regulation in MetS.

Notably, despite these baseline differences, spectral HRV indices exhibited broadly similar short-term responses to glucose ingestion during the OGTT. This suggests that although basal autonomic tone is altered in MetS, acute autonomic adjustments to a metabolic challenge may remain at least partially preserved, albeit from a less favorable autonomic baseline. Given the limited direct literature specifically addressing dynamic HRV responses to OGTT across these phenotypes, this aspect should be interpreted cautiously and warrants further study.

Consistent with the current HRV literature, we adopt the term “relative sympathovagal imbalance” rather than “sympathetic predominance,” acknowledging the physiological complexity and the ongoing debate regarding the interpretation of the LF/HF ratio as a direct index of sympathetic activity [43]. Integrating multidomain HRV descriptors with short RR interval dynamics during the OGTT, we investigated autonomic modulation in participants and its contribution to differentiating participants with MetS, C, and M. The results indicate that time, frequency, and nonlinear domain HRV indices capture complementary facets of autonomic regulation under resting conditions and glucose stimulus induced metabolic stress.

At all OGTT phases, the M group showed the typical autonomic features of endurance-trained subjects, characterized by longer RR intervals, greater global variability (SDNN), and higher short-term parasympathetic indices (RMSSD and SD1). These findings are consistent with other evidence indicating that habitual endurance exercise induces increased vagal activity, retained complexity, and greater autonomic responsiveness [37]. This pattern, by contrast, is consistent with MetS patients, who exhibit consistently shorter RR intervals and overall decreased variability, especially at baseline. This pattern implies sustained vagal withdrawal and very limited autonomic adjustment, and is consistent with an established pattern of studies associating metabolic dysregulation with decreased HRV amplitude and baroreflex function [3,4,10].

These interpretations were also consistent with spectral analysis. MetS participants had reduced VLF and LF power during rest, confirming that metabolic dysfunction impairs slower oscillatory rhythms and disturbs the sympathetic–parasympathetic homeostatic balance. Nonlinear indicators provided insight: diminished fractal structure (DFA-$\alpha_{1}$), lower long-term variability (SD2), and a decline in entropy after 120 min is indicative of a loss of physiological complexity due to glucose exposure. These results corroborate previous work demonstrating that nonlinear HRV indices are sensitive to early autonomic decline and loss of adaptive function in metabolic conditions [9,30].

Our multimodal deep learning results were convergent evidence. An integrative task combining RR temporal structure with HRV descriptions achieved a good discriminative performance in the three class classification tasks. All multimodal architectures had class-wise AUCs between 0.93 and 0.99, with C and M providing the highest class specificity. MetS returned slightly lower values, but similar results that reflect MetS’ rather distinct autonomic phenotype. Normalized confusion matrices showed the CNN–MLP + SVM, as well as CNN + LSTM–MLP + SVM architecture, achieved perfect recall for C and M while correctly identifying 87% of cases of MetS. Distribution of misclassification patterns with respect to C and M was consistent with the expected physiological overlap between MetS and non-MetS autonomic profiles.

The similarity of results between the Softmax and SVM classifier heads indicates that a large majority of discriminative information is encoded in the multimodal encoder. The models were also validated for the inclusion of physiologically interpretable measures such as mean RR, SDNN, LF/HF ratio, fractal exponent, and entropy based on feature importance checks, emphasizing the validity of the learned structures according to bio significance. Combined, these results indicate that integrating RR dynamics with multidomain HRV descriptors not only captures the autonomic regulation of the OGTT more effectively, but also allows for discrimination between metabolic phenotypes beyond either modality alone.

A limitation of this study is that autonomic regulation was assessed indirectly through RR interval dynamics and multidomain HRV indices derived from ECG recordings, without complementary standardized autonomic function tests such as deep breathing, Valsalva maneuver, active standing, or baroreflex sensitivity assessment. This decision was consistent with the specific aim of the study, which was to characterize cardiac autonomic responses to metabolic stimulation during the OGTT using non-invasive ECG-based markers. Therefore, the present findings should be interpreted as indirect evidence of altered cardiac autonomic modulation during OGTT rather than as a comprehensive evaluation of autonomic nervous system function. Future studies may strengthen the physiological interpretation by integrating HRV analysis with formal autonomic testing.

## 5. Conclusions

We show that the integration of multiple-domain HRV descriptors and short-term RR interval dynamics during OGTT captures complementary aspects of autonomic regulation, thus facilitating a clearly discriminative process among individuals with MetS, C, and M, and that resting HRV was particularly revealing in that participants who had MetS consistently demonstrated a reduced global variability, attenuation of low-frequency oscillations, reduced fractal organization, and diminished signal complexity. Together, these observations indicate a chronic impairment in autonomic cardiovascular control linked to metabolic dysregulation.

For the multimodal classifiers tested in this study, performance was impressive, with accuracies near 0.95 and macro AUCs > 0.96. The CNN-MLP model produced the most balanced behaviour amongst all three groups, and the CNN + LSTM-MLP + SVM combination displayed the most obvious separation between classes. Feature relevance analyses bolstered the physiological plausibility of these models, suggesting a consistent integration of variability indexes, spectral characteristics, and nonlinear signals that guided their selection.

In summary, this research suggests new potential for HRV-based multimodal frameworks as a non-invasive yet efficient means for early screening and stratification of MetS. When these physiological descriptors are combined with clinical characteristics such as biochemical markers, anthropometric profiles, and lifestyle indicators, predictive power can be increased, and the potential for more individualized assessment of cardiometabolic risk could be realized.

Future work should consider additional ECG-derived features beyond standard HRV metrics for PR and QT intervals, QTc formulations, ST segment properties, and beat-to-beat morphological variables. Studying the coupling of these variables might provide more subtle autonomic and electrophysiological signatures of the metabolic dysfunction. Future research must also consider model calibration, longitudinal monitoring to evaluate temporal stability, and the inclusion of individuals in the study cohort with isolated metabolic abnormalities, prediabetes, or cardiovascular comorbidities. These processes will make it more generalizable and enable the translation of these HRV-based multimodal systems into clinically relevant, practical use.

## Figures and Tables

**Table 1:** Clinical and biochemical characteristics of the study groups: MetS, Control group (C), and Marathoners (M). Values are presented as median and interquartile range.

Parameter	MetS	C	M	*p*-Value
Age (years)	33.0 (8.5)	27.5 (6.8)	35.0 (11.5)	0.096
BMI (kg/m^2^)	33.0 (5.7)	22.6 (3.9)	20.8 (1.3)	<0.001 *^+^
Systolic BP (mmHg)	137.0 (7.5)	116.0 (9.5)	110.0 (8.0)	<0.001 *^+^
Diastolic BP (mmHg)	88.0 (14.0)	73.0 (8.0)	72.0 (14.0)	0.058
Triglycerides (mg/dL)	194.0 (93.5)	71.0 (30.2)	56.0 (23.0)	<0.001 *^+^
HDL cholesterol (mg/dL)	41.0 (9.5)	46.5 (7.2)	48.0 (8.0)	0.060
Fasting glucose (mg/dL)	103.0 (9.0)	96.5 (5.5)	86.0 (10.0)	<0.001 ^+^
Glucose 120 min (mg/dL)	131.0 (29.0)	96.0 (18.8)	71.0 (21.5)	<0.001 ^+^
Fasting insulin (µUI/mL)	11.0 (6.0)	3.0 (3.0)	2.0 (1.2)	0.0001 *^+^
Insulin 120 min (µUI/mL)	94.0 (88.0)	28.5 (11.2)	16.6 (10.7)	<0.001 *^+^
Glucose AUC_0–120_	18,180.0 (3420.0)	14,220.0 (1631.2)	10,860.0 (1530.0)	<0.001 *^+^
Insulin AUC_0–120_	8415.0 (10,477.5)	3592.5 (750.0)	2655.0 (1583.1)	<0.001 *^+^

* Significant difference between the MetS and the marathoner groups. ^+^ Significant difference between the MetS and control groups.

**Table 2:** Statistics comparison among study groups using time domain HRV parameters. Values are median (IQR), rounded to one decimal.

Parameter	Metabolic Syndrome (MetS)	Marathoner (M)	Control (C)	MetS vs. M	MetS vs. C	M vs. C
				*p*-Value		
$\bar{\mathrm{RR}}$ (ms), 0 min	842.8 (166.8)	1076.9 (244.5)	988.6 (175.9)	<0.001	0.100	0.142
$\bar{\mathrm{RR}}$ (ms), 30 min	879.7 (173.9)	1065.2 (217.3)	986.7 (181.0)	<0.001	0.014	0.382
$\bar{\mathrm{RR}}$ (ms), 60 min	918.2 (195.1)	1039.6 (232.2)	967.6 (101.8)	0.003	0.381	0.108
$\bar{\mathrm{RR}}$ (ms), 90 min	861.0 (208.7)	997.9 (244.7)	959.4 (91.0)	0.010	0.214	0.182
$\bar{\mathrm{RR}}$ (ms), 120 min	876.9 (163.1)	1017.6 (192.2)	932.6 (174.5)	0.008	0.289	0.204
SDNN (ms), 0 min	47.6 (17.0)	98.9 (87.7)	91.0 (52.3)	0.227	0.040	0.308
SDNN (ms), 30 min	54.4 (30.8)	62.5 (44.6)	63.4 (32.9)	0.546	0.824	0.746
SDNN (ms), 60 min	66.0 (38.0)	68.5 (67.1)	69.8 (32.3)	0.431	0.721	0.803
SDNN (ms), 90 min	65.2 (26.1)	69.5 (45.9)	64.7 (18.3)	0.151	0.824	0.224
SDNN (ms), 120 min	64.3 (20.1)	77.3 (72.1)	81.4 (38.9)	0.209	0.114	0.608
RMSSD (ms), 0 min	37.3 (32.7)	54.7 (41.4)	34.6 (27.0)	0.587	0.901	0.712
RMSSD (ms), 30 min	34.0 (23.5)	34.9 (38.4)	42.6 (26.9)	0.325	0.524	0.798
RMSSD (ms), 60 min	43.7 (32.5)	50.1 (47.0)	44.5 (25.6)	0.563	0.924	0.632
RMSSD (ms), 90 min	40.1 (24.6)	31.0 (38.5)	38.7 (23.3)	0.701	0.984	0.744
RMSSD (ms), 120 min	34.7 (19.9)	46.9 (38.9)	43.2 (24.8)	0.692	0.743	0.984
SDSD (ms), 0 min	37.4 (32.8)	54.8 (41.5)	34.7 (27.0)	0.594	0.922	0.708
SDSD (ms), 30 min	34.0 (23.5)	34.9 (38.5)	42.7 (27.0)	0.332	0.514	0.803
SDSD (ms), 60 min	43.8 (32.5)	50.2 (47.1)	44.6 (25.6)	0.572	0.931	0.637
SDSD (ms), 90 min	40.1 (24.6)	31.0 (38.6)	38.7 (23.3)	0.716	0.987	0.755
SDSD (ms), 120 min	34.7 (19.9)	46.9 (39.0)	43.3 (24.9)	0.701	0.726	0.996
pNN50 (%), 0 min	19.3 (34.7)	29.8 (37.4)	12.4 (27.9)	0.927	1.000	0.908
pNN50 (%), 30 min	10.7 (23.5)	11.3 (37.4)	21.4 (27.5)	0.543	0.510	0.941
pNN50 (%), 60 min	24.7 (28.5)	27.5 (43.2)	21.3 (25.6)	0.632	0.904	0.624
pNN50 (%), 90 min	18.0 (25.5)	7.9 (38.2)	17.0 (22.4)	0.987	1.000	1.000
pNN50 (%), 120 min	12.7 (19.7)	13.4 (28.6)	19.4 (18.8)	0.784	0.896	0.976

Values shown in red indicate statistically significant differences.

**Table 3:** Statistical comparison among study groups using frequency-domain HRV parameters. Values are presented as median (IQR).

Parameter	Metabolic Syndrome (MetS)	Marathoner (M)	Control (C)	MetS vs. M	MetS vs. C	M vs. C
				*p*-Value		
VLF (ms^2^), 0 min	2.71 (1.27)	11.11 (27.87)	11.30 (16.12)	0.036	0.016	0.586
VLF (ms^2^), 30 min	4.34 (8.41)	3.03 (10.49)	4.06 (6.91)	0.542	0.785	0.785
VLF (ms^2^), 60 min	6.68 (6.22)	4.90 (19.85)	5.03 (8.11)	0.349	0.737	0.615
VLF (ms^2^), 90 min	4.83 (5.10)	6.15 (13.83)	6.31 (3.85)	0.075	0.706	0.224
VLF (ms^2^), 120 min	4.42 (2.19)	8.01 (45.91)	12.06 (16.42)	0.065	0.063	0.834
LF (ms^2^), 0 min	3.10 (1.96)	5.49 (12.39)	7.34 (9.60)	0.453	0.047	0.189
LF (ms^2^), 30 min	4.66 (4.18)	4.40 (5.54)	5.40 (5.97)	0.472	0.989	0.529
LF (ms^2^), 60 min	5.00 (6.00)	5.39 (11.90)	7.36 (6.52)	0.317	0.386	0.978
LF (ms^2^), 90 min	4.82 (4.98)	8.55 (10.70)	5.71 (6.05)	0.223	0.685	0.494
LF (ms^2^), 120 min	5.18 (5.10)	3.82 (16.98)	7.84 (6.34)	0.472	0.214	0.548
HF (ms^2^), 0 min	1.54 (2.23)	3.09 (3.25)	1.76 (2.59)	0.585	0.807	0.807
HF (ms^2^), 30 min	1.19 (1.65)	1.81 (3.26)	2.41 (2.48)	0.950	0.780	0.823
HF (ms^2^), 60 min	2.53 (2.71)	3.14 (3.92)	2.13 (2.59)	0.876	0.769	0.665
HF (ms^2^), 90 min	2.07 (1.87)	0.97 (3.33)	2.04 (2.27)	0.876	0.989	0.878
HF (ms^2^), 120 min	1.51 (1.77)	2.04 (2.61)	2.24 (4.44)	0.975	0.586	0.605
LF/HF, 0 min	1.86 (1.30)	1.50 (3.55)	4.50 (6.39)	0.595	0.008	0.028
LF/HF, 30 min	2.57 (1.36)	3.22 (2.57)	2.20 (1.73)	0.696	0.438	0.261
LF/HF, 60 min	2.43 (1.58)	3.80 (7.94)	3.65 (1.68)	0.482	0.206	0.525
LF/HF, 90 min	2.78 (1.93)	3.29 (4.51)	2.58 (3.17)	0.184	0.883	0.298
LF/HF, 120 min	2.69 (1.54)	3.77 (3.50)	3.75 (4.27)	0.206	0.339	0.861

Values shown in red indicate statistically significant differences.

**Table 4:** Statistical comparison among study groups using nonlinear HRV parameters. Values are presented as median (IQR), rounded to one decimal place.

Parameter	Metabolic Syndrome (MetS)	Marathoner (M)	Control (C)	MetS vs. M	MetS vs. C	M vs. C
				*p*-Value		
DFA-$\alpha_{1}$, 0 min	1.1 (0.3)	1.3 (0.2)	1.3 (0.3)	0.839	0.017	0.028
DFA-$\alpha_{1}$, 30 min	1.3 (0.2)	1.4 (0.4)	1.2 (0.2)	0.888	0.243	0.196
DFA-$\alpha_{1}$, 60 min	1.3 (0.2)	1.2 (0.3)	1.3 (0.2)	0.685	0.224	0.394
DFA-$\alpha_{1}$, 90 min	1.3 (0.2)	1.3 (0.3)	1.3 (0.3)	0.248	0.511	0.706
DFA-$\alpha_{1}$, 120 min	1.2 (0.2)	1.4 (0.3)	1.4 (0.2)	0.303	0.184	0.685
SD1 (ms), 0 min	26.4 (23.2)	38.7 (29.3)	24.5 (19.1)	0.563	0.928	0.670
SD1 (ms), 30 min	24.0 (16.6)	24.7 (27.2)	30.1 (19.0)	0.295	0.489	0.807
SD1 (ms), 60 min	30.9 (23.0)	35.4 (33.2)	31.5 (18.1)	0.628	0.950	0.620
SD1 (ms), 90 min	28.3 (17.4)	21.9 (27.3)	27.3 (16.5)	0.651	0.983	0.670
SD1 (ms), 120 min	24.5 (14.0)	33.1 (27.5)	30.6 (17.6)	0.662	0.655	0.955
SD2 (ms), 0 min	63.1 (18.4)	134.1 (115.5)	120.8 (70.7)	0.164	0.022	0.291
SD2 (ms), 30 min	73.9 (40.8)	76.1 (59.3)	85.1 (43.3)	0.639	0.900	0.769
SD2 (ms), 60 min	88.0 (47.5)	84.3 (87.2)	93.7 (40.6)	0.472	0.727	0.769
SD2 (ms), 90 min	87.7 (35.3)	96.2 (59.3)	89.1 (24.3)	0.078	0.732	0.216
SD2 (ms), 120 min	87.1 (26.7)	104.2 (98.1)	109.3 (56.1)	0.164	0.082	0.620
SD1/SD2 (%), 0 min	0.36 (0.14)	0.29 (0.14)	0.25 (0.06)	0.160	0.003	0.081
SD1/SD2 (%), 30 min	0.31 (0.08)	0.31 (0.11)	0.34 (0.13)	0.950	0.467	0.434
SD1/SD2 (%), 60 min	0.35 (0.09)	0.33 (0.18)	0.29 (0.12)	0.617	0.451	0.759
SD1/SD2 (%), 90 min	0.32 (0.10)	0.25 (0.12)	0.34 (0.20)	0.200	0.748	0.410
SD1/SD2 (%), 120 min	0.35 (0.09)	0.26 (0.07)	0.27 (0.08)	0.122	0.090	0.753
SampEn, 0 min	1.5 (0.2)	1.5 (0.5)	1.3 (0.4)	0.563	0.087	0.232
SampEn, 30 min	1.4 (0.3)	1.5 (0.4)	1.5 (0.4)	0.390	0.438	0.994
SampEn, 60 min	1.6 (0.2)	1.6 (0.6)	1.4 (0.3)	0.901	0.418	0.356
SampEn, 90 min	1.5 (0.4)	1.3 (0.3)	1.5 (0.5)	0.142	0.685	0.364
SampEn, 120 min	1.5 (0.3)	1.3 (0.2)	1.2 (0.4)	0.041	0.024	0.670
ApEn, 0 min	2.33 (0.12)	2.33 (0.31)	2.30 (0.17)	0.303	0.442	0.878
ApEn, 30 min	2.36 (0.21)	2.37 (0.20)	2.33 (0.11)	0.606	0.238	0.472
ApEn, 60 min	2.34 (0.12)	2.39 (0.10)	2.34 (0.13)	0.435	0.727	0.295
ApEn, 90 min	2.31 (0.20)	2.33 (0.15)	2.44 (0.16)	0.803	0.171	0.111
ApEn, 120 min	2.41 (0.14)	2.40 (0.12)	2.39 (0.08)	0.408	0.553	0.883

Values shown in red indicate statistically significant differences.

**Table 5:** Architecture and training parameters of the multimodal models.

Parameter	Value
RR input length	256 intervals
HRV feature dimension	32
CNN filters	64, 128
Kernel sizes	5, 3
Pooling size	2
RR embedding dimension	64
HRV embedding dimension	32
Fusion embedding dimension	96
Fusion strategy	Late fusion (concatenation)
LSTM units	64
MLP neurons	64, 32
Dropout rates	0.2 (CNN), 0.3 (MLP)
Classifier	Softmax/Linear SVM
Optimizer	Adam
Learning rate	$10^{-3}$
Batch size	32
Epochs (max)	100

**Table 6:** Global performance of the three multimodal models for three class classification (MetS, control, and marathoner).

Model	Accuracy	F1 Macro	Balanced Accuracy	Cohen Kappa	MCC	AUC Macro
CNN–MLP (Softmax)	0.95	0.95	0.96	0.92	0.93	0.97
CNN–MLP + SVM	0.93	0.92	0.93	0.89	0.90	0.96
CNN + LSTM–MLP + SVM	0.92	0.92	0.92	0.88	0.88	0.97

**Table 7:** Per class performance (precision, recall, and F1 score) of the three multimodal models in the test set.

Model	Class	Precision	Recall	F1 Score
CNN–MLP (Softmax)	Control (C)	0.91	1.00	0.95
	Marathoner (M)	0.94	1.00	0.97
	Metabolic syndrome (MetS)	1.00	0.87	0.93
CNN–MLP + SVM	Control (C)	0.91	1.00	0.95
	Marathoner (M)	0.94	1.00	0.97
	Metabolic syndrome (MetS)	1.00	0.87	0.93
CNN+LSTM–MLP + SVM	Control (C)	0.89	0.80	0.84
	Marathoner (M)	0.94	1.00	0.97
	Metabolic syndrome (MetS)	0.87	0.87	0.87

**Table 8:** Top HRV parameters ranked by relative importance in the multimodal models. Only parameters included in the HRV statistical tables were considered.

HRV Parameter	Relative Importance
$\bar{\mathrm{RR}}$	0.110
SDNN	0.095
LF/HF	0.080
SD1/SD2	0.080
*S* (Poincaré area)	0.075
SampEn	0.070
DFA-$\alpha_{1}$	0.065
HF	0.060
LF	0.058
VLF	0.055
RMSSD	0.050
SDSD	0.048
SD2	0.045
SD1	0.042
pNN50	0.040

## Data Availability

The original data presented in the study are openly available in Zenodo at https://zenodo.org/records/19407654 (DOI: 10.5281/zenodo.19407654).

## Abbreviations

The following abbreviations are used in this manuscript:

ApEn	Approximate Entropy
AUC	Area Under the Curve
BMI	Body Mass Index
BP	Blood Pressure
CNN	Convolutional Neural Network
DFA-$\alpha_{1}$, DFA-$\alpha_{2}$	Detrended Fluctuation Analysis Exponents
ECG	Electrocardiogram
FNR	False Negative Rate
FPR	False Positive Rate
HF	High-Frequency Power
HRV	Heart Rate Variability
IQR	Interquartile Range
LF	Low-Frequency Power
LSTM	Long Short-Term Memory
MCC	Matthews Correlation Coefficient
MetS	Metabolic Syndrome
MLP	Multilayer Perceptron
NPV	Negative Predictive Value
OGTT	Oral Glucose Tolerance Test
PPV	Positive Predictive Value
RMSSD	Root Mean Square of Successive Differences
ROC	Receiver Operating Characteristic
SampEn	Sample Entropy
SD1, SD2	Poincaré Plot Descriptors
SDNN	Standard Deviation of NN Intervals
SDSD	Standard Deviation of Successive Differences
SVM	Support Vector Machine
VLF	Very Low-Frequency Power

## References

[1] Wagner P., Strodthoff N., Bousseljot R., Kreiseler D., Lunze F., Samek W., Schaeffter T. (2020). PTB-XL: A Large Publicly Available Electrocardiography Dataset. Sci. Data.

[2] Sun J., Shen H., Qu Q., Sun W., Kong X. (2021). The Application of Deep Learning in Electrocardiogram: Where We Came From and Where We Should Go?. Int. J. Cardiol..

[3] Ma Y., Tseng P., Ahn A., Wu M., Ho Y., Chen M., Peng C. (2017). Cardiac Autonomic Alteration and Metabolic Syndrome: An Ambulatory ECG-Based Study in a General Population. Sci. Rep..

[4] Ortiz-Guzmán J., Mollà-Casanova S., Serra-Año P., Arias-Mutis O., Calvo C., Bizy A., Alberola A., Chorro F., Zarzoso M. (2023). Short-Term Heart Rate Variability in Metabolic Syndrome: A Systematic Review and a Meta-Analysis. J. Clin. Med..

[5] Perpiñán G., Severeyn E., Wong S., Caicedo A., Briceño J., Díaz M. (2019). Cardiac autonomic modulation in response to a glucose stimulus. Med. Biol. Eng. Comput..

[6] Goulopoulou S., Baynard T., Franklin R., Carhart R., Weinstock R., Kanaley J. (2010). Exercise training improves cardiovascular autonomic modulation in response to glucose ingestion in obese adults with and without type 2 diabetes. Metabolism.

[7] Jagannathan R., Neves J., Dorcely B., Chung S., Tamura K., Rhee M., Bergman M. (2020). The Oral Glucose Tolerance Test: 100 Years Later. Diabetes Metab. Syndr. Obes. Targets Ther..

[8] Stumvoll M., Mitrakou A., Pimenta W., Jenssen T., Yki-Järvinen H., Van Haeften T., Gerich J. (2000). Use of the oral glucose tolerance test to assess insulin release and insulin sensitivity. Diabetes Care.

[9] Zamora-Justo J., Campos-Aguilar M., Beas-Jara M., Galván-Fernández P., Ponciano-Gómez A., Sigrist-Flores S., Jiménez-Flores R., Muñoz-Diosdado A. (2025). Utility of nonlinear analysis of heart rate variability in early detection of metabolic syndrome. Front. Physiol..

[10] Azulay N., Olsen R., Nielsen C., Stubhaug A., Jenssen T., Schirmer H., Frigessi A., Rossel L., Tronstad C. (2022). Reduced heart rate variability is associated with metabolic syndrome components and diabetes in the sixth Tromsø Study 2007–2008. Sci. Rep..

[11] Hannun A., Rajpurkar P., Haghpanahi M., Tison G., Bourn C., Turakhia M., Ng A. (2019). Cardiologist-Level Arrhythmia Detection and Classification in Ambulatory ECG Using a Deep Neural Network. Nat. Med..

[12] Chen Y., Liu C., Tseng V., Hu Y., Chen S. Large-scale 12-lead ECG classification using deep learning. Proceedings of the IEEE EMBS International Conference on Biomedical & Health Informatics (BHI).

[13] Çınar A., Tuncer S. (2021). Classification of normal sinus rhythm, abnormal arrhythmia, and congestive heart failure ECG signals using LSTM and hybrid deep neural networks CNN–SVM. Comput. Methods Biomech. Biomed. Eng..

[14] Bhatia S., Pandey S., Kumar A., Alshuhail A. (2022). Classification of Electrocardiogram Signals Based on Hybrid Deep Learning Models. Sustainability.

[15] Madan P., Singh V., Singh D., Diwakar M., Pant B., Kishor A. (2022). A Hybrid Deep Learning Approach for ECG-Based Arrhythmia Classification. Bioengineering.

[16] Hwang S., Kwon N., Lee D., Kim J., Yang S., Youn I., Moon H., Sung J., Han S. (2023). A Multimodal Fatigue Detection System Using sEMG and IMU Signals with a Hybrid CNN–LSTM–Attention Model. Sensors.

[17] Anbarasi A., Ravi T. (2023). Detection and classification of arrhythmia type using hybrid model of LSTM with convolutional neural network. Appl. Nanosci..

[18] Cheng J., Zou Q., Zhao Y. (2021). Clasificación de señales de ECG basada en CNN profunda y BiLSTM. BMC Med. Inform. Decis. Mak..

[19] de Faria E., de Vilhena E., Miosso C. (2025). Metabolic Syndrome Detection Based on Classification of Electrocardiography Signals. Sensors.

[20] Ribeiro A., Ribeiro M., Paixao G., Oliveira D., Gomes P., Canazart J., Ferreira M., Oliveira J., Silva R., Santos D. (2020). Automatic Diagnosis of the 12-Lead ECG Using a Deep Neural Network. Nat. Commun..

[21] (2001). Expert Panel on Detection, Evaluation, and Treatment of High Blood Cholesterol in Adults. Executive Summary of the Third Report of the NCEP Expert Panel on Detection, Evaluation, and Treatment of High Blood Cholesterol in Adults. JAMA.

[22] Hawley J., Lessard S. (2008). Exercise training-induced improvements in insulin action. Acta Physiol..

[23] Bird S., Hawley J. (2017). Update on the effects of physical activity on insulin sensitivity in humans. BMJ Open Sport Exerc. Med..

[24] Ledezma C., Severeyn E., Perpiñán G., Altuve M., Wong S. A new on-line electrocardiographic records database and computer routines for data analysis. Proceedings of the 36th Annual International Conference of the IEEE Engineering in Medicine and Biology Society (EMBC).

[25] Pan J., Tompkins W. (1985). A Real-Time QRS Detection Algorithm. IEEE Trans. Biomed. Eng..

[26] Dotsinsky I. (2007). Advanced methods and tools for ECG analysis. Biomed. Eng. Online.

[27] Task Force of the European Society of Cardiology and the North American Society of Pacing and Electrophysiology (1996). Heart Rate Variability: Standards of Measurement, Physiological Interpretation, and Clinical Use. Circulation.

[28] Shaffer F., Ginsberg J. (2017). An Overview of Heart Rate Variability Metrics and Norms. Front. Public Health.

[29] Chen D., Huang H., Bao X., Pan J., Li Y. (2023). An EEG-based attention recognition method: Fusion of time domain, frequency domain, and non-linear dynamics features. Front. Neurosci..

[30] Acharya U., Joseph K., Kannathal N., Lim C., Suri J. (2006). Heart Rate Variability: A Review. Med. Biol. Eng. Comput..

[31] Ullah A., Rehman S., Tu S., Mehmood R., Fawad M., Ehatisham-ul haq M. (2021). A Hybrid Deep CNN Model for Abnormal Arrhythmia Detection Based on Cardiac ECG Signal. Sensors.

[32] Nahiduzzaman M., Islam M., Islam S., Goni M., Anower M., Kwak K. (2021). Hybrid CNN-SVD Based Prominent Feature Extraction and Selection for Diabetic Retinopathy Classification Using Extreme Learning Machine Algorithm. IEEE Access.

[33] Rai H., Chatterjee K. (2022). Hybrid CNN–LSTM Deep Learning Model and Ensemble Technique for Automatic Detection of Myocardial Infarction Using ECG Big Data. Appl. Intell..

[34] Sokolova M., Lapalme G. (2009). A systematic analysis of performance measures for classification tasks. Inf. Process. Manag..

[35] Powers D. (2011). Evaluation: From Precision, Recall and F-measure to ROC, Informedness, Markedness and Correlation. J. Mach. Learn. Technol..

[36] Moreno M., Sarmiento B., Moran D., Casares F., Suarez O. (2023). Automated computer-aided diagnosis of COVID-19 and pneumonia based on chest X-ray images using deep learning: Classification and segmentation. Modern Computational Techniques for Engineering Applications.

[37] Aubert A., Seps B., Beckers F. (2003). Heart Rate Variability in Athletes. Sport. Med..

[38] Richman J., Moorman J. (2000). Physiological time-series analysis using approximate entropy and sample entropy. Am. J. Physiol.-Heart Circ. Physiol..

[39] Stuckey M., Tulppo M., Kiviniemi A., Petrella R. (2014). Metabolic syndrome and heart rate variability: A systematic review. Diabetes/Metab. Res. Rev..

[40] Buchheit M., Gindre C. (2006). Cardiac parasympathetic regulation: Respective associations with cardiorespiratory fitness and training load. Am. J. Physiol.-Heart Circ. Physiol..

[41] Plews D., Laursen P., Kilding A., Buchheit M. (2013). Training adaptation and heart rate variability in elite endurance athletes: Opening the door to effective monitoring. Sport. Med..

[42] Grassi G., Dell’Oro R., Quarti-Trevano F., Scopelliti F., Seravalle G., Paleari F., Mancia G. (2005). Neuroadrenergic and reflex abnormalities in patients with metabolic syndrome. Diabetologia.

[43] Billman G. (2013). The LF/HF ratio does not accurately measure cardiac sympatho-vagal balance. Front. Physiol..

